# Obituary for Professor Dr. Jan Evangelista Dyr

**DOI:** 10.3390/metabo11040243

**Published:** 2021-04-15

**Authors:** Leona Mášová-Chrastinová, John W. Weisel

**Affiliations:** 1 Department of Biochemistry, Institute of Hematology and Blood Transfusion, U Nemocnice 2094/1, 128 20 Prague, Czech Republic; Leona.Chrastinova@uhkt.cz; 2 Department of Cell and Developmental Biology, Perelman School of Medicine, University of Pennsylvania, Philadelphia, PA 19104, USA

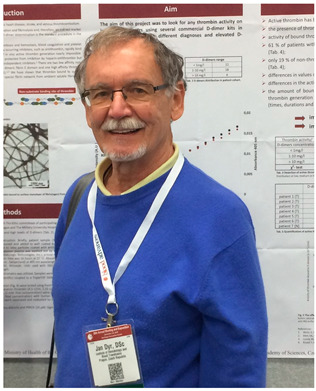



Prof. Dr. Jan Evangelista Dyr

Jan Dyr, our cherished colleague, passed away on 22 January 2021 after a short course of the COVID-19 disease.

Jan was born on 24 September 1946 in Prague, Czechoslovakia. He obtained his undergraduate degree in Macromolecular Chemistry from the University of Chemistry and Technology in Prague in 1969. Then, he earned a Ph.D. in Physical Chemistry in 1978 from Charles University and a DrSc. in Chemistry in 1994 from the Academy of Sciences of the Czech Republic.

In 1984 he became the Head of the Department of Biochemistry at the Institute of Hematology and Blood Transfusion. In 2004 he was appointed as the Head of the Research Division and in the same year he became the Deputy Director of this institute. From 2007 to 2009 he acted as the statutory director of the Institute of Hematology and Blood Transfusion. He also was an educator, lecturing from 2003 at the Medical Faculty of Charles University as an Associate Professor and from 2005 at the University of Chemistry and Technology as a Professor.

Jan completed two internships as a research scientist in the team of Professor Birger Blombäck at the Karolinska Institutet in Stockholm in 1981 and 1984. After these visits his country’s repressive government did not allow him to travel to the West. However, he went to the Institute of Biochemistry in Kiev, Ukraine, to meet with Prof. Leonid Medved. During his stay with Prof. Medved he met another visitor—Prof. John Weisel. After the Velvet Revolution, these international collaborations expanded, with many of his students going abroad to study at Weisel´s laboratory and other institutions.

Jan led a research team in the field of hemostasis and thrombosis for many years. The fibrinogen molecule fascinated him from the beginning of his career. Together with his longtime colleague and friend, Dr. Jiri Suttnar, he discovered that the fibrinopeptides are split off fibrinogen in a different manner when fibrinogen is adherent on a surface, in comparison to when it is in solution. Later, he and Jiri studied the structure and interaction properties of fibrinogen in many unique situations, for example in fibrinogen modified by oxidation, and the influence of oxidative stress on blood platelets. Their main tools were chromatographic and electrophoretic techniques. Jan was the leader of the team producing monoclonal antibodies against fibrinogen that became commercially available with his influence. Due to his love of fibrinogen and his outstanding studies in this field, Jan established scientific collaborations all over the world.

With his great knowledge in the field, he became an editor of the journal “Thrombosis Research” during the years 1989–1993 and 1998–2003. Recently, he used theoretical methods of structural biology, such as molecular modeling and molecular dynamics simulations, primarily to study fibrinogen and its interactions with other molecules in the blood. Using these techniques, he studied the effects of post-translational modifications and mutations on the structure and functions of fibrinogen, described the interactions of fibrinogen with thrombin and hemostatic snake venoms, and predicted the structure of parts of fibrinogen that could not be determined experimentally.

Jan Dyr was always looking for new horizons for his research and used the latest techniques to fulfill his goals. For example, he used molecular biology and mass spectrometry methods to discover hypofibrinogenemias, as well as many congenital and acquired dysfibrinogenemias. In the last eight years his group at the Department of Biochemistry identified 46 new cases of acquired afibrinogenemia and three cases of congenital afibrinogenemia. Thanks to his long-term cooperation and friendship with Prof. Jiri Homola, Jan contributed to the design of protein biochips for surface plasmon resonance, an optical detection method. His specific goal was to evaluate the sensitivity and specificity of the surface plasmon resonance method for the observation and quantification of individual proteins and protein complexes related to oncohematological diseases and to identify disease-associated mechanisms by validating microRNA and mRNA profiling data of patients and healthy individuals at the protein level using surface plasmon resonance biosensors. These activities illustrate Jan’s efforts to broaden his research on fibrinogen and include other plasma proteins. By analysis of subproteomes in human plasma and blood cells, he looked for differences in the expression of proteins and their post-translational modifications to better understand the pathogenesis of oncohematological and cardiovascular diseases. In these innovative projects, he cooperated closely with Prof. Pavel Klener, Martin Maly M.D., etc., and many institutions, such as the Medical Faculty of Charles University, the Military University Hospital, the University of Chemistry and Technology, the Institute of Photonics and Electronics, the Institute of Physics, and the Institute of Macromolecular Chemistry, etc., in Prague.

The key methodologies of his later research were quite broad ranging, including proteomics, interactomics, complexomics, and metabolomics. One of his last research projects was on the presence of active thrombin on fibrin D-dimer. Bound thrombin is less susceptible to inhibition by the heparin/antithrombin complex in comparison with free thrombin, and results in thromboses. The ongoing pandemic of COVID-19 has shown us how problematic the prediction of thrombotic complications in patients with multiple organ deterioration and failure can be. Jan authored about 170 publications in peer-reviewed journals and 9 national patents, and his works generated more than 1000 citations (Web of Science). In 2019, he was elected Chairman of the Czech Society for Thrombosis and Hemostasis.

Jan made everyone around him a better scientist because of both his knowledge and wisdom and the power of his personality. He was a man of many ideas and generously shared them. He had strong opinions and unselfishly and gently provided constructive criticism that often went to the heart of the matter and helped to advance the science. He had a very distinctive sense of humor and loved Monty Python. Jan was a very cultivated and moral man, who was devoted to his family and always stood by his students and the values of science. With his passing, a painful gap will remain in every respect, both for his family, friends, and colleagues, and for his field.

